# Vitamin D supplementation in COVID-19: A friend or foe?

**DOI:** 10.17179/excli2020-2696

**Published:** 2020-08-17

**Authors:** Asieh Mansour, Mohammad Reza Mohajeri-Tehrani, Sayed Mahmoud Sajjadi-Jazi

**Affiliations:** 1 Endocrinology and Metabolism Research Institute (EMRI), Tehran University of Medical Sciences, Tehran, Iran; 2 Cell Therapy and Regenerative Medicine Research Center, Endocrinology and Metabolism Molecular-Cellular Sciences Institute, Tehran University of Medical Sciences, Tehran, Iran

## ⁯⁯⁯

***Dear Editor,***

Coronavirus disease of 2019 (COVID-19), a novel coronavirus infection, is fast becoming the epidemic of the 21^st ^century. Individuals with COVID-19 are at an increased risk of developing lower respiratory tract disorders, such as pneumonia, acute respiratory distress syndrome (ARDS), and even death (Ramanathan et al., 2020[13]). To stop or slow the development of COVID-19-associated complications, new preventive or therapeutic options must be developed. 

Recently, the use of vitamin D as a preventive/therapeutic intervention against COVID-19 infection has received much attention (Daneshkhah et al., 2020[2]). A trial registered with ClinicalTrial.gov proposes to evaluate the role of vitamin D in hard endpoints related to COVID-19's deleterious consequences (NCT04334005). However, no published clinical trial is yet available for vitamin D administration in COVID-19 patients.

Vitamin D is well-known for its action on the respiratory system and its deficiency is linked to the progression of pneumonia (Wang et al., 2017[17]; Zhou et al., 2019[19])*. *It has been hypothesized that low levels of vitamin D could contribute to influenza virus infection (Beard et al., 2011[1]). According to a recent meta-analysis of 25 trials (11,321 participants), based on a study of a variety of populations, the risk of acute respiratory tract infection was found to be decreased with vitamin D supplementation (Martineau et al., 2017[10]). In addition, vitamin D, via its receptor (VDR), can be important for providing an effective innate immune response, particularly by increasing the production of cathelicidine (antimicrobial peptides) (Beard et al., 2011[1]). Vitamin D also has beneficial effects on adaptive immunity by regulating B and T cells proliferation and function (Liu et al., 2006[8]; Moise and Balescu-Arion, 2020[11]). Finally, vitamin D has been established as a negative endocrine regulator of the renin angiotensin system (RAS) and a long-term vitamin D deficiency has been shown to up-regulate the RAS activity. RAS plays an important pathophysiological role in the etiology of major diseases, including lung injury induced by sepsis (Kong et al., 2013[5]; Li, 2018[7]). In contrast, some studies have suggested that vitamin D supplementation does not prevent the respiratory tract infection; it may even be harmful, especially in those who are not vitamin D deficient and/or those who receive bolus doses (Lehouck et al., 2012[6]; Manaseki-Holland et al., 2012[9]; Remmelts et al., 2013[14]). For instance, three independent case-control studies of more than 130,000 participants, mostly older individuals, have shown that vitamin D supplementation does not change or may even increase the risk of developing pneumonia (Remmelts et al., 2013[14]). COVID-19 binds with their target cells through angiotensin-converting enzyme 2 (ACE2), which is expressed in various tissues and cell types, including pulmonary alveolar epithelial cells, small intestinal enterocytes, renal tubular cells and endothelial cells of blood vessels (Fang et al., 2020[4]; Sun et al., 2020[16]). Consequently, the up-regulation of ACE2 can facilitate the infection with COVID-19. Xu et al. have suggested that the expression of ACE2 is substantially increased in the rat model of acute lung injury, which is treated with calcitriol in a concentration-dependent manner (Xu et al., 2017[18]). Vitamin D also suppresses tumor necrosis factor alpha converting enzyme (TACE) (Dusso et al., 2010[3]), which is involved in the proteolytic cleavage and the degradation of ACE2 (Pedersen et al., 2015[12]; Suh et al., 2020[15]). If these data could be confirmed, vitamin D, especially in those who also use other ACE2-inducing drugs such as ACE inhibitors, angiotensin II type-I receptor blockers (ARBs), thiazolidinediones and ibuprofen (Fang et al., 2020[4]), may increase the risk of developing severe and fatal COVID-19 infection by the up-regulation of ACE2. These data, therefore, cast doubt on the beneficial effects of vitamin D for the prevention and treatment of respiratory tract infections, including COVID-19.

To summarize, first, the review of literature on the beneficial effects of vitamin D against COVID-19 infection leads to some doubts and controversies; therefore, well-designed clinical trials are needed to assess the potential risk-benefit of vitamin D supplementation for the prevention and/or treatment of COVID-19 infection. Second, although appropriate vitamin D doses may show some benefits in reducing the respiratory tract infections, higher doses may be harmful. Third, limited information is available on the *in vitro *and* in vivo *vitamin D-mediated ACE2 (a functional receptor for COVID-19 infection) metabolism and its interactions with other ACE2-inducing drugs. Finally, until further data regarding the efficacy of vitamin D on the COVID-19 infection is available, clinicians should exercise caution in the overuse of vitamin D, especially in the elderly patients who also use other drugs which may alter ACE2 expression. Until further studies can fill these gaps, the single daily dose of 800-1000 IU vitamin D may be sufficient to meet the needs of most of the population. 

## Competing interests

None of the authors have any conflicts of interest or financial ties to disclose.

## Funding

Not applicable.
